# Differential Response of Stro-1^+^ and Stro-1^−^ Shed to Er,Cr:YSGG Laser Stimulation: Viability, Matrix Production and Lineage Commitment

**DOI:** 10.3390/jfb17030138

**Published:** 2026-03-10

**Authors:** Zornitsa Mihaylova, Marina Miteva, Emilia Karova, Natalia Grancharova, Violeta Dogandzhiyska, Mirela Marinova-Takorova, Krasimir Hristov, Vanyo Mitev, Evgeniy Aleksiev, Dimitar Kosturkov, Nadezhda Mitova, Irina Tsenova-Ilieva, Nikolay Ishkitiev

**Affiliations:** 1 Research Institute of Innovative Medical Science, Medical University Sofia, 1000 Sofia, Bulgaria; 2 Department of Dental, Oral and Maxillofacial Surgery, Faculty of Dental Medicine, Medical University Sofia, 1000 Sofia, Bulgaria; 3 Department of Chemistry and Biochemistry, Medical Faculty, Medical University Sofia, 1000 Sofia, Bulgaria; m.miteva@medfac.mu-sofia.bg (M.M.); nishkitiev@medfac.mu-sofia.bg (N.I.); 4 Department of Conservative Dentistry, Faculty of Dental Medicine, Medical University Sofia, 1000 Sofia, Bulgariav.dogandjiska@fdm.mu-sofia.bg (V.D.); irinatsenova@yahoo.com (I.T.-I.); 5 Department of Pediatric Dentistry, Faculty of Dental Medicine, Medical University Sofia, 1000 Sofia, Bulgaria; n.grancharova@fdm.mu-sofia.bg (N.G.); k.christov@fdm.mu-sofia.bg (K.H.); n.mitova@fdm.mu-sofia.bg (N.M.)

**Keywords:** SHED, STRO-1, Er,Cr:YSGG laser, osteogenic differentiation, collagen synthesis, dental pulp regeneration

## Abstract

Stem cell heterogeneity represents a critical yet underexplored variable in laser-assisted regenerative strategies. While photobiomodulation has been shown to influence mesenchymal stem cell (MSC) behavior, it remains unclear whether stem cell maturation status modulates responsiveness to Er,Cr:YSGG irradiation. This study investigated the differential response of magnetically separated STRO-1^+^ and STRO-1^−^ SHED subpopulations to low-power Er,Cr:YSGG laser stimulation (0.10 W and 0.25 W), focusing on viability, extracellular matrix production, and lineage commitment. STRO-1^+^ cells comprised 13.4% ± 1.2% of the total Stem Cells from Human Exfoliated Deciduous teeth (SHED) population. Laser exposure did not impair metabolic activity in either subpopulation. Collagen synthesis demonstrated a power- and time-dependent increase, with maximal enhancement observed in STRO-1^+^ cells at 0.25 W after 7 days. Laser irradiation selectively promoted osteogenic differentiation, as evidenced by increased alkaline phosphatase (ALP) expression at 0.10 W and enhanced mineral deposition, while chondrogenic potential remained unaffected and adipogenesis was reduced following 0.10 W exposure. These findings suggest that ALP expression is temporally and power-dependently modulated during osteogenic progression. Overall, Er,Cr:YSGG photobiomodulation does not uniformly affect heterogeneous SHED populations but modulates lineage allocation and extracellular matrix deposition in a maturation- and power-dependent manner. Integrating stem cell subpopulation selection with laser-based bioactivation may represent a strategy to refine regenerative endodontic and biomaterial-guided therapies.

## 1. Introduction

The application of laser systems in dentistry has expanded significantly, and they are now widely integrated into both clinical practice and laboratory research. Among these, the erbium, chromium-doped yttrium-scandium-gallium-garnet (Er,Cr:YSGG) laser has gained particular attention due to its selective absorption by water and hydroxyapatite [1]. This property makes it highly effective for procedures involving both soft and hard dental tissues. Additionally, its precision and ability to remove tissue with minimal heat-induced damage have established it as an essential tool in restorative dentistry [2].

In recent years, laser technologies have become increasingly attractive for regenerative therapies in dentistry, particularly in regenerative endodontics. Lasers are utilized for precise tissue removal, minimization of thermal damage, and enhancement of tissue healing. Growing evidence suggests that laser systems, including Er,Cr:YSGG, may play a role in stimulating the regeneration of dental pulp, especially in primary teeth [3]. The use of lasers for regenerative endodontic therapies is a promising direction, as they may help facilitate the repair and regeneration of dental tissues following pulp injury or disease [4,5].

One of the most promising applications of the Er,Cr:YSGG laser in regenerative endodontics involves the stimulation of dental pulp regeneration, particularly in primary teeth, through Stem Cells from Human Exfoliated Deciduous Teeth (SHED). These multipotent stem cells possess strong regenerative potential and can differentiate into odontoblasts, osteoblasts, and other mesenchymal cell types [6]. In addition, other dental-derived stem cell populations have been widely studied [7,8] due to their accessibility and high regenerative capacity. SHED, in particular, have garnered significant interest due to their ability to promote pulp tissue regeneration [9].

Although SHED exhibit strong regenerative potential, it is important to note that the complete regeneration of primary tooth pulp depends on several factors, such as the developmental stage of the tooth and the degree of root resorption. SHED, however, stand out as a promising cell population due to their high proliferation rate, which makes them especially useful in tissue engineering applications [10]. They secrete a wide range of growth factors and cytokines that support angiogenesis, immune modulation, and tissue repair [11]. Furthermore, SHED show strong adaptability to various microenvironments, allowing them to survive and function effectively in damaged pulp tissue. Their low immunogenicity makes them suitable candidates for both autologous and potentially allogenic therapeutic applications [12]. Thus, SHED represent one of the most promising sources of stem cells for regenerative dental therapies.

STRO-1 is widely used as a marker of immature mesenchymal stem/progenitor cells and has been associated with enhanced clonogenicity, proliferative capacity, and metabolic activity [13]. Importantly, photobiomodulation exerts its biological effects primarily through mitochondrial activation and modulation of cellular metabolism [14]. Therefore, it is biologically plausible that SHED subpopulations at different maturation states, defined by STRO-1 expression, may respond differently to Er,Cr:YSGG laser irradiation. Segregation of SHED into STRO-1^+^ and STRO-1^−^ fractions thus enables a more mechanistic evaluation of laser-mediated cellular responses.

Although photobiomodulation influences MSC behavior, it remains unclear whether stem cell maturation status modulates the biological response to Er,Cr:YSGG irradiation. Limited information is available regarding whether SHED subpopulations defined by STRO-1 expression exhibit differential responsiveness to Er,Cr:YSGG laser stimulation. Addressing this gap may refine regenerative strategies by integrating selective stem cell enrichment with laser-based bioactivation approaches.

The aim of this study was to investigate whether magnetically separated STRO-1^+^ and STRO-1^−^ SHED subpopulations exhibit differential biological responses to Er,Cr:YSGG laser irradiation at 0.10 W and 0.25 W. Previous in vitro studies have demonstrated that low-power Er,Cr:YSGG laser irradiation can modulate dental pulp cell metabolism without inducing cytotoxic effects [3]. Therefore, the present study employed low irradiation powers (0.10 W and 0.25 W) to evaluate photobiomodulatory effects on distinct SHED subpopulations. A broad range of cellular responses were assessed, including metabolic activity (MTT), early and late mineralization markers, adipogenic and chondrogenic differentiation potential, and collagen synthesis. These experimental results aim to clarify the potential of Er,Cr:YSGG irradiation to modulate the biological behavior of distinct SHED subsets, and to evaluate its influence on stem cell-based regenerative endodontic strategies.

## 2. Materials and Methods

### 2.1. Isolation and Culture of SHED

Exfoliated deciduous teeth (*n* = 3) were collected from pediatric patients treated at the Faculty of Dental Medicine, Medical University of Sofia, Bulgaria, with written informed consent from parents or guardians in accordance with institutional ethical standards and the Declaration of Helsinki. The study was approved by the Medical Science Council of the Medical University of Sofia (approval number: 4770/11 December 2018). SHED were isolated following established protocols [15]. Teeth were washed in Phosphate-Buffered Saline (PBS; Sigma-Aldrich, St. Louis, MO, USA), and dental pulp was carefully extracted under sterile conditions. Pulp tissue was enzymatically digested using collagenase type I and dispase (both Sigma-Aldrich), filtered, and cultured in Dulbecco’s Modified Eagle Medium (DMEM; Invitrogen, Eugene, OR, USA) supplemented with 10% fetal bovine serum (FBS), (Invitrogen), 100 U/mL penicillin, 100 μg/mL streptomycin, and 0.25 μg/mL amphotericin B (all Invitrogen) at 37 °C in a humidified 5% CO_2_ atmosphere. Medium was replaced every 2–3 days. Cells were expanded until 85–90% confluence before passaging. SHED at passages 3–5 were used for all experiments (the workflow is given in Figure S1).

### 2.2. Magnetic Separation of STRO-1^+^ SHED

When cultures reached >1 × 10^5^ cells per T25 flask (TPP^®^, Trasadingen, Switzerland), magnetic-activated cell sorting (MACS) was performed following our previous protocols [16,17]. MACS was performed using the following reagents and protocol: Primary Antibody: Mouse monoclonal anti-STRO-1 (Abcam, Tokyo, Japan), (Catalogue #ab29263, Clone STRO-1; 10 μL per 10^7^ cells), Secondary Reagent: Goat anti-mouse IgG MicroBeads (Miltenyi Biotec GmbH, Bergisch Gladbach, Germany), (Catalogue #130-113-019; 20 μL per 10^7^ cells), Separation Column: MiniMACS^TM^ Separator (Miltenyi Biotec GmbH, Catalogue #130-042-102). Cells were detached with trypsin–EDTA (Lonza, Verviers, Belgium), washed twice in cold PBS supplemented with 2% FBS and 2 mM EDTA (MACS buffer), and incubated with primary anti-STRO-1 antibody (1:10 dilution in MACS buffer) for 15 min at 4 °C. After washing, cells were incubated with goat anti-mouse IgG MicroBeads (1:5 dilution in MACS buffer) for 15 min at 4 °C. Labeled cells were passed through a MiniMACS^TM^ Separator: unlabeled STRO-1^−^ cells flowed through into a collection tube, while magnetically labeled STRO-1^+^ cells were retained on the column and subsequently eluted with fresh medium containing 2% FBS. STRO-1^+^ and STRO-1^−^ fractions were expanded separately in DMEM at 5 × 10^2^ cells/cm^2^ until sufficient numbers were obtained for experiments. Purity of STRO-1^+^ cells was ensured by MACS and confirmed in the present study by immunocytochemical staining for STRO-1.

### 2.3. Quantitative Evaluation of STRO-1^+^ Cells

Aliquots from each isolated SHED fraction were washed with PBS supplemented with 2% FBS, and total cell numbers were determined using a hemocytometer. Cell viability was assessed by trypan blue exclusion and calculated as follows:

Viability (%) = (number of viable cells/total number of cells) × 100.

The yield of the STRO-1^+^ fraction was expressed both as the total number of viable cells recovered per flask and as the percentage of viable cells relative to the initial input population. All cell counts were performed in triplicate. Immunocytochemical staining was conducted to confirm STRO-1 expression and to evaluate cellular morphology.

### 2.4. Er,Cr:YSGG Laser Application

A Waterlase^®^ C100 (Biolase, Forest, CA, USA) laser with wavelength 2780 nm, pulse repetition rate 15 Hz, and pulse duration 60 µs was used. The laser tip had a diameter of 400 µm and a length of 9 mm. Laser energy was delivered in non-contact mode, perpendicular to the well surface, at an approximate 1 mm distance, in a continuous horizontal sweeping motion for 10 s. Cells were maintained in standard culture medium with serum during irradiation, and the laser tip was not immersed; irradiation was performed through the transparent bottom of the 96-well plate. This setup was designed to deliver sub-ablative laser exposure, minimizing thermal or mechanical stress to the cells.

Experimental groups (*n* = 3 biological replicates, each in triplicate technical repeats) and corresponding laser parameters are summarized in Table 1.

Laser irradiation was performed in non-contact mode at an approximate 1 mm distance. Although temperature was not directly measured, the applied parameters correspond to sub-ablative photobiomodulation conditions, and no morphological changes or viability loss were observed, indicating negligible thermal effects.

### 2.5. MTT Cell Viability Assay

The purpose is to evaluate the effect of laser irradiation on SHED viability. Cell viability was evaluated using the MTT Cell Proliferation Kit (Abcam). SHED were seeded in 96-well flat-bottom plates (Costar, Corning, New York, NY, USA) at a density of 5 × 10^3^ cells per well in 100 μL of culture medium and allowed to attach overnight. Laser irradiation was performed directly in the same 96-well plates as described above. MTT assays were carried out at 24 h and 72 h following laser treatment. Subsequently, 10 μL of MTT reagent (0.5 mg/mL) was added to each well and incubated for 4 h at 37 °C. Formazan crystals were solubilized overnight in 100 μL solubilization buffer, and absorbance measured at 550–600 nm with reference > 650 nm (Varioskan, Thermo Fisher, Vantaa, Finland). Results are expressed as mean ± SD, calculated from three biological replicates, each performed in triplicate, and normalized to the non-irradiated control group.

### 2.6. Immunocytochemical Analysis of STRO-1 and ALP Expression in Magnetically Separated SHED Subpopulations

STRO-1^+^ and STRO-1^−^ SHED were seeded at a density of 5 × 10^3^ cells per well in 24-well culture plates on glass coverslips (Menzel-Gläser, Braunschweig, Germany) and cultured for 24 h. Cells were fixed with 4% paraformaldehyde (Sigma-Aldrich, St. Louis, MO, USA) in PBS for 15 min at room temperature, then permeabilized sequentially using 0.05% Tween-20 (Sigma-Aldrich) in PBS for 10 min, followed by 0.05% Triton X-100 (Sigma-Aldrich) for 30 min. Cells were then blocked with 1% bovine serum albumin (BSA; Sigma-Aldrich) in PBS for 30 min at room temperature. Primary antibodies were diluted in 1% BSA in PBS and applied as follows: Mouse monoclonal anti-STRO-1 (Abcam, Cambridge, UK; Catalogue #ab29263, Clone STRO-1; dilution 1:200) for 2 h at room temperature, or Mouse monoclonal anti-alkaline phosphatase (ALP) (Sigma-Aldrich, Catalogue #A4630, Clone 8; dilution 1:100) for 2 h at room temperature. After three washes with PBS (5 min each), secondary antibody was applied: Goat Anti-Mouse IgG (H + L) Alexa Fluor^®^ 568 conjugate (Abcam, Catalogue #ab175701; dilution 1:500 in 1% BSA) for 1 h at room temperature in darkness. Nuclei were counterstained with DAPI (dilution 1:5000, included in mounting medium). Coverslips were mounted on glass slides using ProLong^®^ Gold Antifade Mountant with DAPI (Thermo Fisher Scientific, Waltham, MA, USA; Catalogue #P36941) and stored at 4 °C in darkness until imaging (within 24 h of mounting). Fluorescence images were acquired using an In Cell Analyzer 6000 imaging system (GE Healthcare, Pittsburgh, PA, USA) with the following settings: STRO-1 channel (yellow): Alexa Fluor 568 excitation (575 nm), emission filter 600–680 nm; DAPI channel (blue): excitation 405 nm, emission filter 440–480 nm; Image acquisition: pixel size 0.64 μm; 8-bit depth; gain 500–700 (optimized to prevent saturation). At least three independent fields per well, three wells per group were assessed.

Image analysis was performed using In Cell Analyzer Workstation software (version 3.7.3; GE Healthcare, Chicago, IL, USA). Morphological analysis and verification of STRO-1 separation fidelity were performed by visual inspection of immunofluorescence signal intensity and cellular morphology. For semi-quantitative ALP analysis, ALP activity was analyzed in standard cell culture medium and conditions to evaluate the basal osteogenic potential of STRO-1^+^ and STRO-1^−^ SHED, without prior osteogenic induction. Fluorescence intensity was measured in at least 50 cells per well using automated image analysis with background subtraction, and mean pixel intensity (arbitrary units) was calculated and compared across groups.

### 2.7. Total Collagen Synthesis Test

The main aim is to assess extracellular matrix production after laser treatment. SHED were seeded in 24-well plates at a density of 2 × 10^4^ cells per well in 500 μL of culture medium and allowed to attach overnight. Laser irradiation was applied as described above, and cells were maintained under standard culture conditions for 3 or 7 days prior to supernatant collection. Total collagen was quantified using the Sircol^TM^ Collagen Assay (Biocolor Ltd., Belfast, UK) according to the manufacturer’s instructions. Briefly, 100 μL of cell culture supernatant was mixed with 1 mL of dye reagent and incubated for 30 min at room temperature. The resulting collagen–dye complex was centrifuged at 12,000 rpm for 10 min, washed with acid–salt reagent, and solubilized in 250 μL of alkali reagent. Absorbance was measured at 555 nm using a Varioskan microplate reader (Thermo Fisher Scientific, USA). Collagen synthesis was evaluated at days 3 and 7 post-laser irradiation.

### 2.8. In Vitro Differentiation of Er,Cr:YSGG Laser Irradiated STRO-1^+^ and STRO-1^−^ SHED

The main purpose is to evaluate the effect of 0.10 W laser exposure on multilineage differentiation. Therefore, STRO-1^+^ and STRO-1^−^ SHED were seeded and subjected to Er,Cr:YSGG laser irradiation at 0.10 W. Osteogenic, chondrogenic, and adipogenic differentiation was induced as follows: 1. Osteogenic—DMEM supplemented with 10% FBS, 50 μg/mL ascorbic acid, 10 mM β-glycerophosphate, and 100 nM dexamethasone; medium refreshed every three days. Mineralized nodule formation was assessed after 21 days using Alizarin Red S (Sigma-Aldrich); 2. Chondrogenic: high-glucose DMEM with 1% insulin–transferrin–selenium (ITS), 10 ng/mL TGF-β, 50 μg/mL ascorbic acid, and 100 nM dexamethasone; evaluated after 21 days using Alcian Blue staining (Sigma-Aldrich); 3. Adipogenic: DMEM with 10% FBS, 0.5 mM IBMX, 1 μM dexamethasone, 10 μg/mL insulin, and 100 μM indomethacin; medium renewed every 2–3 days, lipid droplet formation assessed after 14 days using Oil Red O (Sigma-Aldrich). Differentiation was assessed using quantitative dye extraction assays.

For the analysis, bound dyes were extracted as follows: Alizarin Red S with 10% acetic acid, Oil Red O with isopropanol, and Alcian Blue with 10% acetic acid or 0.1 N HCl. Absorbance was measured using a microplate reader (Varioskan, Thermo Fisher) at 570 nm, 510 nm, and 620 nm, respectively, and values were normalized to the total number of cells per well to allow standardized comparison of differentiation efficiency among STRO-1^+^ and STRO-1^−^ SHED populations.

### 2.9. Statistical Analysis

All experiments were performed using cells derived from three independent donors (*n* = 3 biological replicates). For each donor, all experimental conditions were performed in triplicate wells, which were treated as technical replicates. For statistical analysis, values from technical replicates were first averaged per donor, and these donor-level means were used for all subsequent statistical comparisons. Data normality and homogeneity of variances were assessed using the Shapiro–Wilk test. Since all datasets were normally distributed, parametric statistical analysis was applied. Differences among experimental groups were determined using one-way ANOVA followed by Tukey’s post hoc test for multiple comparisons. Differences were considered statistically significant at *p* < 0.05. All analyses were performed using IBM SPSS Statistics software (version 31.0.1.0; IBM Corp., Armonk, NY, USA).

## 3. Results

### 3.1. Quantitative Assessment of STRO-1^+^ SHED Cells

Quantitative analysis following MACS revealed that 13.4% ± 1.21% of the total SHED population was positive for STRO-1. Cell viability remained consistently high following separation, with no significant differences observed between independent replicates.

The yield of the STRO-1^+^ fraction, defined as the proportion of viable magnetically retained cells relative to the total number of SHED subjected to MACS, corresponded closely to the calculated percentage of STRO-1–expressing cells within the initial population.

**Figure 1:**
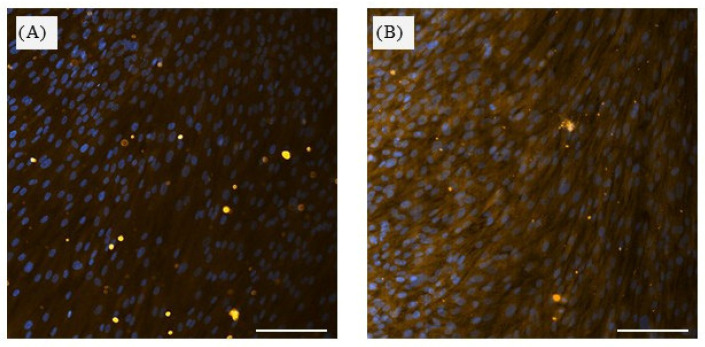
Immunocytochemical characterization of STRO-1^+^ and STRO-1^−^ SHED fractions following magnetic separation. (**A**) STRO-1^−^ fraction after magnetic-activated cell sorting, showing DAPI-stained nuclei (blue) and absence of STRO-1 immunofluorescence (yellow). (**B**) STRO-1^+^ fraction, showing DAPI-stained nuclei (blue) and STRO-1 immunoreactivity (yellow) in positively selected cells. Scale bars (white lines): 50 μm; magnification ×20.

The efficiency of the magnetic separation was further validated by immunocytochemical analysis. Strong STRO-1 immunoreactivity was observed exclusively in the positively selected cell fraction, whereas no detectable signal was present in STRO-1^−^ cells, confirming effective segregation of the two subpopulations. In addition, STRO-1^+^ cells exhibited a fibroblast-like morphology characteristic of mesenchymal stem cells (Figure 1).

### 3.2. MTT Cell Viability

**Figure 2:**
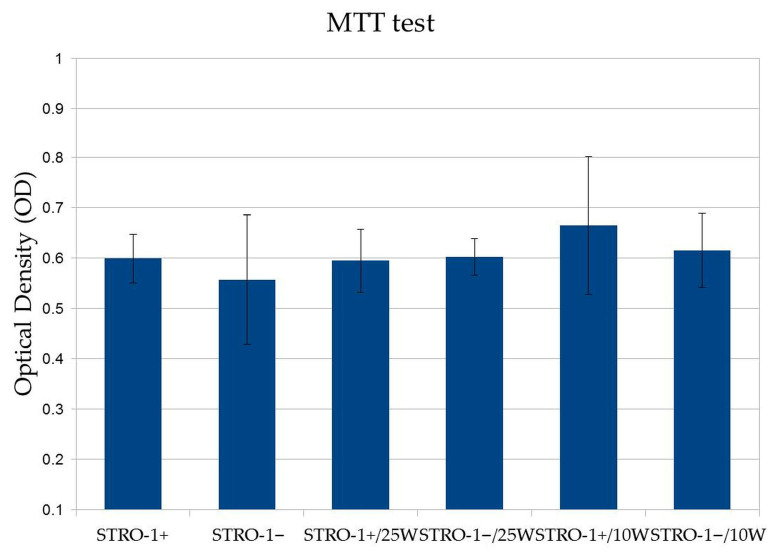
MTT assay of STRO-1^+^ and STRO-1^−^ SHED subpopulations 72 h after Er,Cr:YSGG laser irradiation at 0.10 W and 0.25 W. No statistically significant differences in metabolic activity were observed between subpopulations or treatment groups (*p* > 0.05, One-way ANOVA with Tukey’s post hoc test). Data are presented as mean ± SD from three independent biological replicates (*n* = 3).

MTT analysis performed 72 h after laser irradiation demonstrated no statistically significant differences in metabolic activity between STRO-1^+^ and STRO-1^−^ SHED subpopulations across all experimental conditions (Figure 2).

Under control conditions, mean absorbance values were 0.599 ± 0.048 for STRO-1^+^ cells and 0.557 ± 0.128 for STRO-1^−^ cells. Similarly, irradiation at 0.25 W resulted in comparable viability between STRO-1^+^ (0.594 ± 0.063) and STRO-1^−^ (0.602 ± 0.036) cells. Exposure to 0.10 W produced a comparable pattern, with absorbance values of 0.665 ± 0.137 for STRO-1^+^ cells and 0.615 ± 0.074 for STRO-1^−^ cells.

Overall, Er,Cr:YSGG laser irradiation at both tested power settings did not adversely affect cell viability in either SHED subpopulation within the first 72 h following exposure.

### 3.3. Total Collagen Synthesis

**Figure 3:**
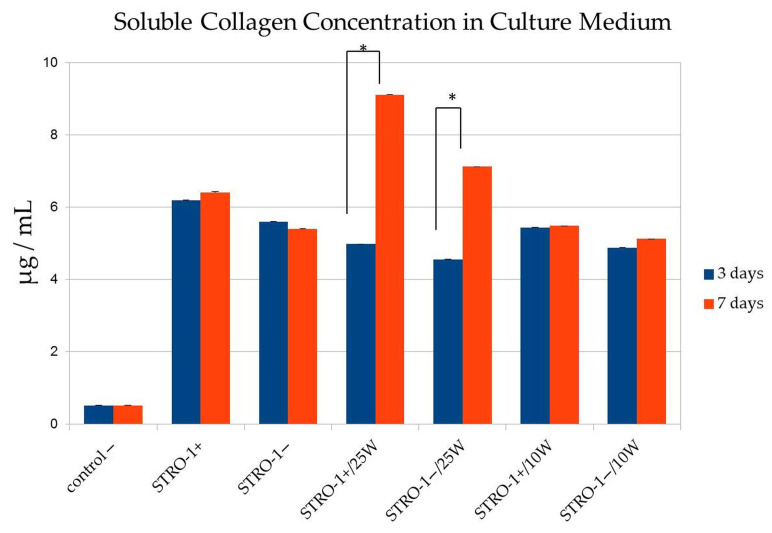
Total collagen synthesis in STRO-1^+^ and STRO-1^−^ SHED subpopulations at days 3 and 7 following Er,Cr:YSGG laser irradiation. Data represent mean ± SD from three independent biological replicates (*n* = 3), each performed in triplicate as technical repeats. Statistical analysis: one-way ANOVA with Tukey’s post hoc test. * Asterisk (*p* < 0.05) indicates significant difference between day 3 and day 7 within the 0.25 W treatment groups for both subpopulations. No significant differences were detected between day 3 and day 7 in non-irradiated controls or 0.10 W groups.

Total collagen synthesis was measured in STRO-1^+^ and STRO-1^−^ SHED populations at days 3 and 7 following Er,Cr:YSGG laser irradiation at 0 W (control), 0.10 W, or 0.25 W (Figure 3). At day 3, collagen levels were similar across all groups: STRO-1^+^, 0.329 ± 0.001; STRO-1^−^, 0.303 ± 0.001, with slight decreases observed in the 0.25 W groups (STRO-1^+^, 0.278 ± 0.001; STRO-1^−^, 0.259 ± 0.001). By day 7, both STRO-1^+^ and STRO-1^−^ populations irradiated at 0.25 W showed a significant increase in collagen synthesis compared with day 3 (STRO-1^+^, 0.452 ± 0.001; STRO-1^−^, 0.367 ± 0.001; *p* < 0.05). No significant changes were observed between days 3 and 7 in non-irradiated controls or in 0.10 W groups. These results highlight a time- and power-dependent effect of Er,Cr:YSGG laser irradiation on collagen production in both SHED subpopulations.

### 3.4. In Vitro Multilineage Differentiation of Er,Cr:YSGG-Irradiated STRO-1^+^ and STRO-1^−^ SHED

The multilineage differentiation potential of STRO-1^+^ and STRO-1^−^ SHED was assessed following Er,Cr:YSGG laser exposure at 0.10 W. Osteogenic differentiation was clearly enhanced in both subpopulations, as indicated by increased Alizarin Red S staining and elevated calcium deposition relative to non-irradiated controls (*p* < 0.05).

Chondrogenic differentiation, evaluated via Alcian Blue staining for sulfated glycosaminoglycans, remained unchanged, with no significant differences detected between irradiated and control cells (*p* > 0.05).

Adipogenic differentiation was reduced in both STRO-1^+^ and STRO-1^−^ SHED following laser exposure, as indicated by decreased Oil Red O staining and lipid accumulation compared to controls (*p* < 0.05).

**Figure 4:**
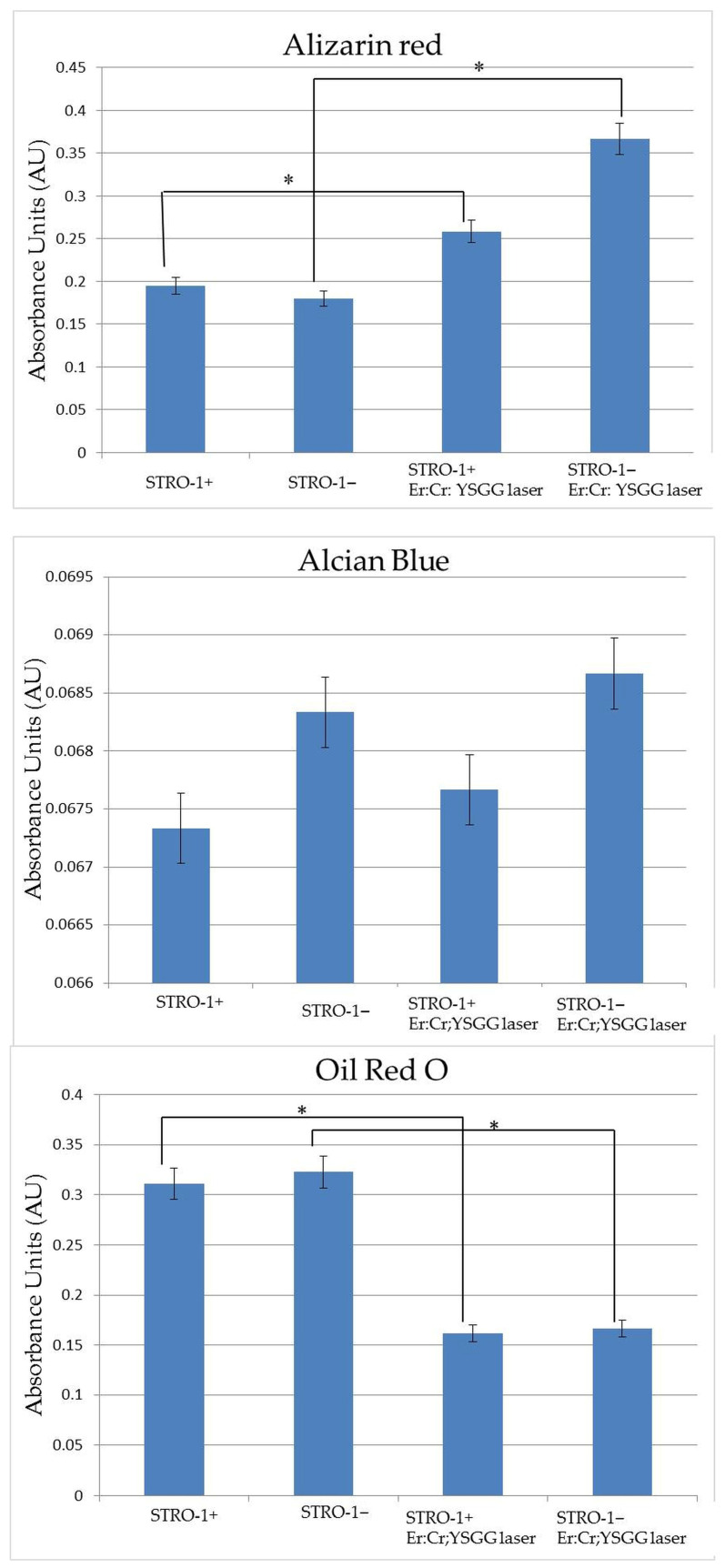
Multilineage differentiation of STRO-1^+^ and STRO-1^−^ SHED following 0.10 W Er,Cr:YSGG laser exposure. Alizarin Red S staining (calcium deposition) for osteogenic differentiation assessment; Alcian Blue staining (sulfated glycosaminoglycans) for chondrogenic differentiation assessment; Oil Red O staining (lipid accumulation) for adipogenic differentiation assessment. Data represent mean ± SD from three independent biological replicates (*n* = 3), each performed in triplicate as technical repeats. Quantitative analysis: absorbance values were normalized to total cell number and compared via one-way ANOVA with Tukey’s post hoc test. * Asterisk (*p* < 0.05) indicates statistical significance between laser-irradiated and non-irradiated control groups.

These findings suggest that low-power (0.10 W) Er,Cr:YSGG irradiation selectively enhances osteogenic differentiation without affecting chondrogenesis, while concurrently inhibiting adipogenic differentiation in both SHED subpopulations. The observed effects were consistent across three independent experiments, supporting the reproducibility of these laser-mediated differentiation responses (Figure 4). Representative images of stained cells before and after laser irradiation are given in Figures S2 and S3, corresponding to the presented quantitative data.

### 3.5. Immunocytochemical Analysis of ALP Expression in Separated SHED Subpopulations

**Figure 5:**
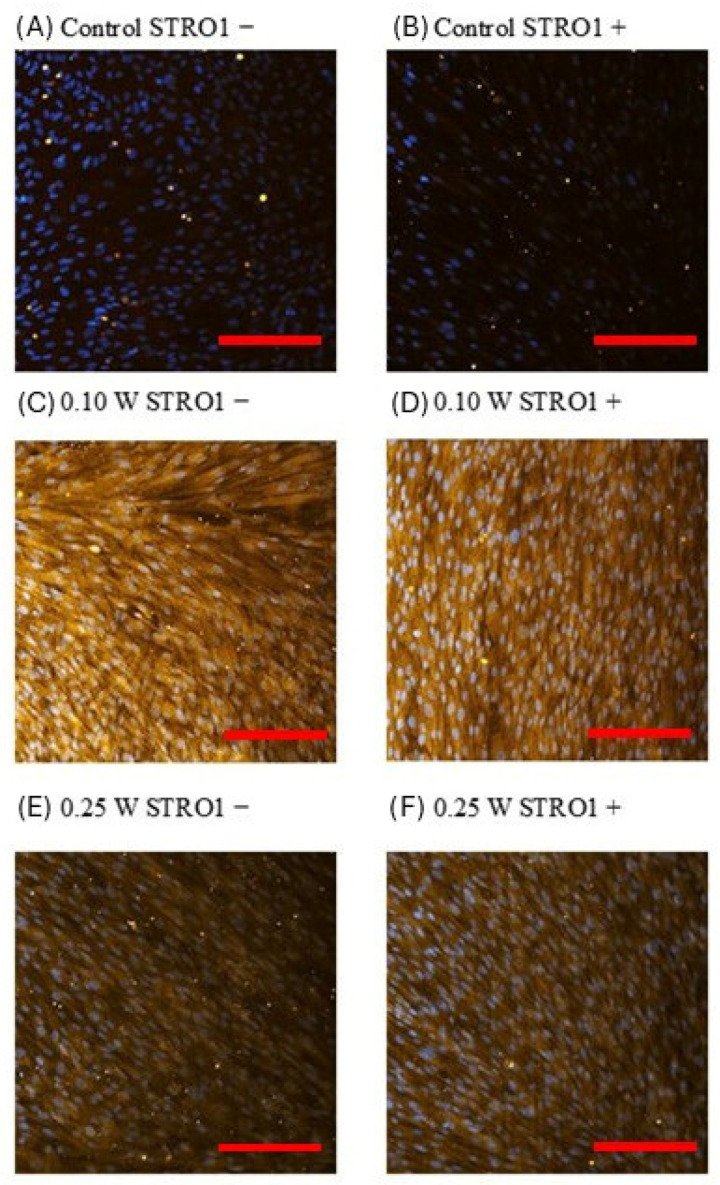
Immunocytochemical analysis of alkaline phosphatase (ALP) expression in magnetically separated STRO-1^+^ and STRO-1^−^ SHED subpopulations following Er,Cr:YSGG laser irradiation. Immunofluorescence images show yellow fluorescence corresponding to Alexa Fluor 568–conjugated anti-ALP antibody (early osteogenic marker) and blue fluorescence indicating DAPI-stained nuclei. (**A**) Control STRO-1^−^ cells (0 W); (**B**) Control STRO-1^+^ cells (0 W); (**C**) STRO-1^−^ cells irradiated at 0.10 W; (**D**) STRO-1^+^ cells irradiated at 0.10 W; (**E**) STRO-1^−^ cells irradiated at 0.25 W; (**F**) STRO-1^+^ cells irradiated at 0.25 W. A visibly stronger ALP immunofluorescence signal is observed in the irradiated groups, particularly at 0.10 W, compared with the corresponding controls. Scale bars (red lines): 20 μm; magnification ×20.

Semi-quantitative immunocytochemical analysis of ALP expression at day 3 post-laser irradiation revealed an increase in ALP immunofluorescence in both STRO-1^+^ and STRO-1^−^ SHED following Er,Cr:YSGG laser irradiation compared with the non-irradiated controls (Figure 5). The strongest ALP signal was observed at 0.10 W, whereas cells irradiated at 0.25 W showed a slightly lower expression compared with the 0.10 W group, but still higher than that of the control cells.

ALP is a well-established marker associated with the initiation and early stages of mineralization [18], and its upregulation suggests that Er,Cr:YSGG photobiomodulation may stimulate early osteogenic activity in SHED subpopulations. The data supporting the statistical analysis are provided as Supplementary Materials.

## 4. Discussion

In this study, we investigated the effects of Er,Cr:YSGG laser treatment on SHED subpopulations separated based on STRO-1 expression, focusing on viability, extracellular matrix production, and multilineage differentiation. Our findings demonstrate that laser treatment can selectively enhance osteogenic differentiation while preserving cell viability and specifically modulating lineage commitment.

The decision to analyze magnetically separated STRO-1^+^ and STRO-1^−^ SHED subpopulations was based on the well-established role of STRO-1 as a marker of immature mesenchymal stem cells (MSC). STRO-1 is a typical marker of immature MSC, commonly used in combination with other markers such as CD146, CD105, and CD271 to better define stem/progenitor populations [13]. In this study, STRO-1 was used to enrich primitive SHED subpopulations, acknowledging that it is not a sole definitive stemness marker [19]. Since SHED represent a highly heterogeneous population, separating them according to STRO-1 expression allowed distinction between a more stem-like subset (STRO-1^+^) and a more differentiated subset (STRO-1^−^). Magnetic-activated cell sorting (MACS) ensured high-purity isolation while preserving cell viability and function, as previously demonstrated for STRO-1^+^ dental pulp cell enrichment using magnetic selection techniques [20]. The magnetic separation approach effectively enriched STRO-1^+^ cells, which constituted 13.4% ± 1.21% of the total SHED population, and immunocytochemical analysis confirmed strong STRO-1 expression in the positive fraction and absence in STRO-1^−^ cells. Although not directly evaluated in this study, previous reports indicate that the proportion of STRO-1^+^ cells may decrease with increasing passage number, suggesting passage-dependent loss of stemness marker expression [21]. High viability across both fractions further confirms that the isolation procedure preserves cellular integrity, enabling reliable functional analyses of the distinct subpopulations.

To date, most studies investigating STRO-1^+^ dental pulp stem cell (DPSCs)s have focused on permanent teeth, where MACS has been employed to isolate primitive stem cell subpopulations for functional analyses [22,23]. While SHED also express STRO-1 and demonstrate a high regenerative potential [3], there is a paucity of studies applying MACS to separate SHED into STRO-1^+^ and STRO-1^−^ subpopulations. This highlights a gap in the literature regarding functional differences between these subsets in deciduous teeth. By magnetically sorting SHED based on STRO-1 expression, our study provides a unique perspective on how Er,Cr:YSGG laser treatment differentially affects primitive versus more differentiated SHED subpopulations. This approach allows a more precise evaluation of laser-induced responses in distinct SHED subpopulations and provides additional information on the functional heterogeneity of these cells in the context of regenerative endodontics.

The MTT assay results show that Er,Cr:YSGG irradiation at both 0.10 W and 0.25 W does not significantly alter mitochondrial metabolic activity in either STRO-1^+^ or STRO-1^−^ SHED populations within the first 72 h post-exposure, indicating preserved cell viability following laser irradiation. It is important to emphasize that MTT is a measure of cellular metabolic activity rather than a direct proliferation assay, and stable mitochondrial activity suggests absence of acute cytotoxicity or metabolic impairment [24]. In support of our findings, prior in vitro studies on mesenchymal stem/stromal cells have reported no reduction in cell viability after photobiomodulation: for example, MSC from dental pulp maintained viability after 3 and 7 days post-irradiation with no significant difference compared to controls [25]; similarly, other studies employing low-level laser/LED protocols documented preserved metabolic activity and cell survival under comparable conditions [26]. Our MTT data support the cytocompatibility of the selected Er,Cr:YSGG parameters, reinforcing that the subsequent increase in collagen production and differentiation markers is unlikely driven by selective cell death or survival bias.

Laser treatment selectively enhanced osteogenic differentiation, demonstrated by increased Alizarin Red staining, while chondrogenic potential assessed by Alcian Blue remained unchanged. In contrast, adipogenic differentiation was reduced, as indicated by decreased Oil Red O accumulation. These findings suggest a shift in the tested cells toward the osteogenic lineage without affecting chondrogenic commitment, consistent with previous reports showing that photobiomodulation can bias MSC differentiation toward osteogenesis while minimally affecting chondrogenic pathways and reducing adipogenesis [27,28].

In this study, untreated SHED subpopulations served as reference controls for lineage-specific differentiation. Standard differentiation protocols for SHED, including the use of positive inducers such as BMP-2, were previously validated [29]. The observed pattern may reflect an intrinsic tendency of more primitive STRO-1^+^ cells to favor osteogenic pathways [30]. Reduced adipogenesis may indicate preferential allocation of cellular resources toward osteogenic differentiation [31], while the unchanged chondrogenic response suggests that STRO-1 expression does not strongly influence chondrogenic commitment under the applied culture conditions [32]. These findings highlight how stem cell maturity may modulate lineage-specific responses and contribute to differential effects of laser treatment on SHED subpopulations.

The increase in ALP expression following Er,Cr:YSGG laser irradiation suggests stimulation of early osteogenic activity, as ALP is associated with the initiation phase of mineralization [33]. The strongest ALP signal was observed at 0.10 W, while cells irradiated at 0.25 W showed slightly lower expression but still higher levels compared with controls. Increased mineral deposition detected by Alizarin Red staining at later stages further supports progression of osteogenic differentiation after laser treatment [34]. Because intermediate time points were not measured, further studies are needed to clarify the temporal dynamics of ALP expression.

Although ALP intensity was normalized to total cell number, slight variations in cell density in the 0.25 W wells may have influenced the relative fluorescence signal. Therefore, this reduction should be interpreted cautiously, and future studies with larger sample sizes and per-cell quantification are needed to confirm these findings.

These results are consistent with previous studies demonstrating that photobiomodulation enhances osteogenic maturation and mineralization. Low-level laser irradiation (635/809 nm) increased mineral deposition in adipose-derived stem cells without sustained ALP elevation [35], while red LED irradiation of periodontal ligament stem cells promoted late-stage osteogenic differentiation without prolonged ALP activity [36]. Similarly, Er:YAG and Er,Cr:YSGG laser treatments enhance osteogenic differentiation in mesenchymal dental stem cells, as shown by increased osteogenic marker expression and mineral deposition [25,37]. Together, these findings indicate that laser photobiomodulation promotes progression toward mineralizing osteoblasts while selectively modulating lineage commitment in MSCs [38].

Our results demonstrate that Er,Cr:YSGG laser irradiation modulates collagen synthesis in both STRO-1^+^ and STRO-1^−^ SHED populations in a power- and time-dependent manner. At day 3, collagen production was comparable between experimental groups, with a slight reduction observed in the 0.25 W irradiated cells, suggesting a transient or delayed response to higher laser energy. By day 7, collagen synthesis markedly increased in both STRO-1^+^ and STRO-1^−^ populations following 0.25 W irradiation, with STRO-1^+^ cells reaching higher absolute levels. ALP activity showed a transient, power-dependent increase, peaking at lower laser settings (0.10 W), coinciding with collagen production trends, which indicates that early osteogenic markers are temporally modulated while matrix deposition is enhanced at later stages. In contrast, collagen production in the 0.10 W groups and non-laser exposed controls remained relatively stable, indicating that lower laser power is insufficient to induce significant changes in extracellular matrix deposition over the examined period.

While STRO-1^+^ cells showed a trend toward slightly higher responsiveness to Er,Cr:YSGG laser treatment in terms of collagen production and osteogenic markers, the observed differences compared to STRO-1^−^ cells were moderate. Both subpopulations exhibited increased collagen synthesis and osteogenic progression, indicating that laser irradiation broadly enhances differentiation. These trends should be interpreted cautiously, and further studies with larger sample sizes are warranted to clarify whether STRO-1^+^ cells consistently exhibit higher sensitivity to photobiomodulation.

These findings suggest that Er,Cr:YSGG treatment at higher power (0.25 W) can enhance extracellular matrix production, potentially contributing to the promotion of osteogenic differentiation and tissue regeneration. The observed increase in collagen aligns with previous reports demonstrating that Er,Cr:YSGG laser irradiation can stimulate fibroblast proliferation and collagen synthesis, supporting tissue repair processes [39] and enhance DNA synthesis, collagen, and procollagen production in vitro [40].

Recent in vitro studies support the notion that photobiomodulation can enhance osteogenic differentiation of dental-derived stem cells and related MSC populations. For example, low-level laser therapy was shown to significantly promote osteogenic differentiation and mineralization in human dental pulp and gingiva-derived MSCs, with upregulation of key osteogenic markers and increased mineral deposition following irradiation at appropriate energy levels [41]. Moreover, systematic evidence indicates that photobiomodulation enhances proliferation and osteogenic outcomes in oral and adipose-derived MSCs, highlighting the potential role of specific energy density parameters in modulating MSC differentiation pathways [42]. These findings align with and help contextualize our results showing that Er,Cr:YSGG laser irradiation at certain power settings selectively promotes osteogenic progression in SHED subpopulations, supporting the therapeutic relevance of laser-based protocols in regenerative endodontic strategies.

Overall, our findings indicate that Er,Cr:YSGG laser irradiation at specific power settings can enhance extracellular matrix production and support osteogenic differentiation of SHED, providing a potential strategy to improve tissue engineering outcomes.

## 5. Conclusions

Er,Cr:YSGG laser irradiation modulates SHED behavior in a maturation- and power-dependent manner. STRO-1^+^ and STRO-1^−^ subpopulations showed enhanced osteogenic differentiation, evidenced by increased ALP expression and mineral deposition, as well as stimulated collagen synthesis, while adipogenic differentiation was reduced. These results indicate selective modulation of lineage commitment. Combining stem cell subpopulation enrichment with controlled laser exposure provides a practical approach to optimize regenerative endodontic strategies, taking into account stem cell heterogeneity for more targeted tissue engineering outcomes.

## Figures and Tables

**Table 1:** Experimental groups, SHED subpopulations, and Er,Cr:YSGG laser parameters. Spot area was calculated from a 400 µm tip diameter. Energy density (fluence) and power density were calculated relative to the effectively irradiated well area due to the continuous sweeping motion of the laser beam. Total energy delivered per well is indicated for each condition. Each group included n = 3 biological replicates, each performed in triplicate as technical repeats.

Group	Subpopulation	Laser Power (W)	Spot Area (mm^2^)	Energy Density (J/cm^2^)	Power Density (W/cm^2^)	Total Energy Per Well (J)
1	STRO-1^+^	0 (control)	0.126	0	0	0
2	STRO-1^−^	0 (control)	0.126	0	0	0
3	STRO-1^+^	0.10	0.126	0.79	7.9	1.0
4	STRO-1^−^	0.10	0.126	0.79	7.9	1.0
5	STRO-1^+^	0.25	0.126	1.97	19.7	2.5
6	STRO-1^−^	0.25	0.126	1.97	19.7	2.5

## Data Availability

The original contributions presented in the study are included in the article/Supplementary Materials, further inquiries can be directed to the corresponding author.

## Supplementary Materials

The following supporting information can be downloaded at: https://www.mdpi.com/article/10.3390/jfb17030138/s1, Supplementary Figure S1—Schematic representation of the experimental workflow. SHED were isolated and subjected to magnetic-activated cell sorting (MACS), followed by laser irradiation under defined parameters, properties assessment and subsequent differentiation assays. The diagram summarizes the sequential steps of the study design. Supplementary Figure S2—non-irradiated. Osteogenic, chondrogenic, and adipogenic differentiation of SHED without Er,Cr:YSGG laser irradiation. Representative images of SHED cultured under standard conditions. Cells were stained with Alizarin Red S (osteogenesis), Alcian Blue (chondrogenesis), and Oil Red O (adipogenesis) to visualize lineage-specific differentiation. These images correspond to the quantitative data presented in the main text. Supplementary Figure S3—irradiated. Osteogenic, chondrogenic, and adipogenic differentiation of SHED after Er,Cr:YSGG laser irradiation. Representative images of SHED exposed Er,Cr:YSGG laser irradiation. Cells were stained with Alizarin Red S (osteogenesis), Alcian Blue (chondrogenesis), and Oil Red O (adipogenesis). These images correspond to the quantitative data presented in the main text and illustrate enhanced differentiation after laser exposure. Supplementary Table S1—Pairwise comparisons of cell intensity between laser doses and STRO-1 subpopulations.

## Abbreviations

The following abbreviations are used in this manuscript:

ALP	Alkaline Phosphatase
ANOVA	Analysis of Variance
ATP	Adenosine Triphosphate
CO_2_	Carbon Dioxide
DAPI	4′,6-Diamidino-2-Phenylindole
DMEM	Dulbecco’s Modified Eagle Medium
DPSCs	Dental Pulp Stem Cells
ECM	Extracellular Matrix
Er,Cr:YSGG	Erbium, Chromium-doped Yttrium-Scandium-Gallium-Garnet Laser
Er:YAG	Erbium-doped Yttrium Aluminum Garnet Laser
FBS	Fetal Bovine Serum
HCl	Hydrochloric Acid
IBMX	3-Isobutyl-1-Methylxanthine
IgG	Immunoglobulin G
ITS	Insulin–Transferrin–Selenium supplement
LED	Light-Emitting Diode
MACS	Magnetic-Activated Cell Sorting
MSC	Mesenchymal Stem/Stromal Cells
MTT	3-(4,5-Dimethylthiazol-2-yl)-2,5-Diphenyltetrazolium Bromide Assay
PBS	Phosphate-Buffered Saline
SD	Standard Deviation
SHED	Stem Cells from Human Exfoliated Deciduous Teeth
SPSS	Statistical Package for the Social Sciences
STRO-1	Mesenchymal stem cell surface marker STRO-1
TGF-β	Transforming Growth Factor Beta
