# Supplementary figures and images for: Differential Response of Stro-1+ and Stro-1− Shed to Er,Cr:YSGG Laser Stimulation: Viability, Matrix Production and Lineage Commitment
Source: J Funct Biomater. 2026 Mar 10;17(3):138. doi: 10.3390/jfb17030138 (PMC13027365; doi:10.3390/jfb17030138)

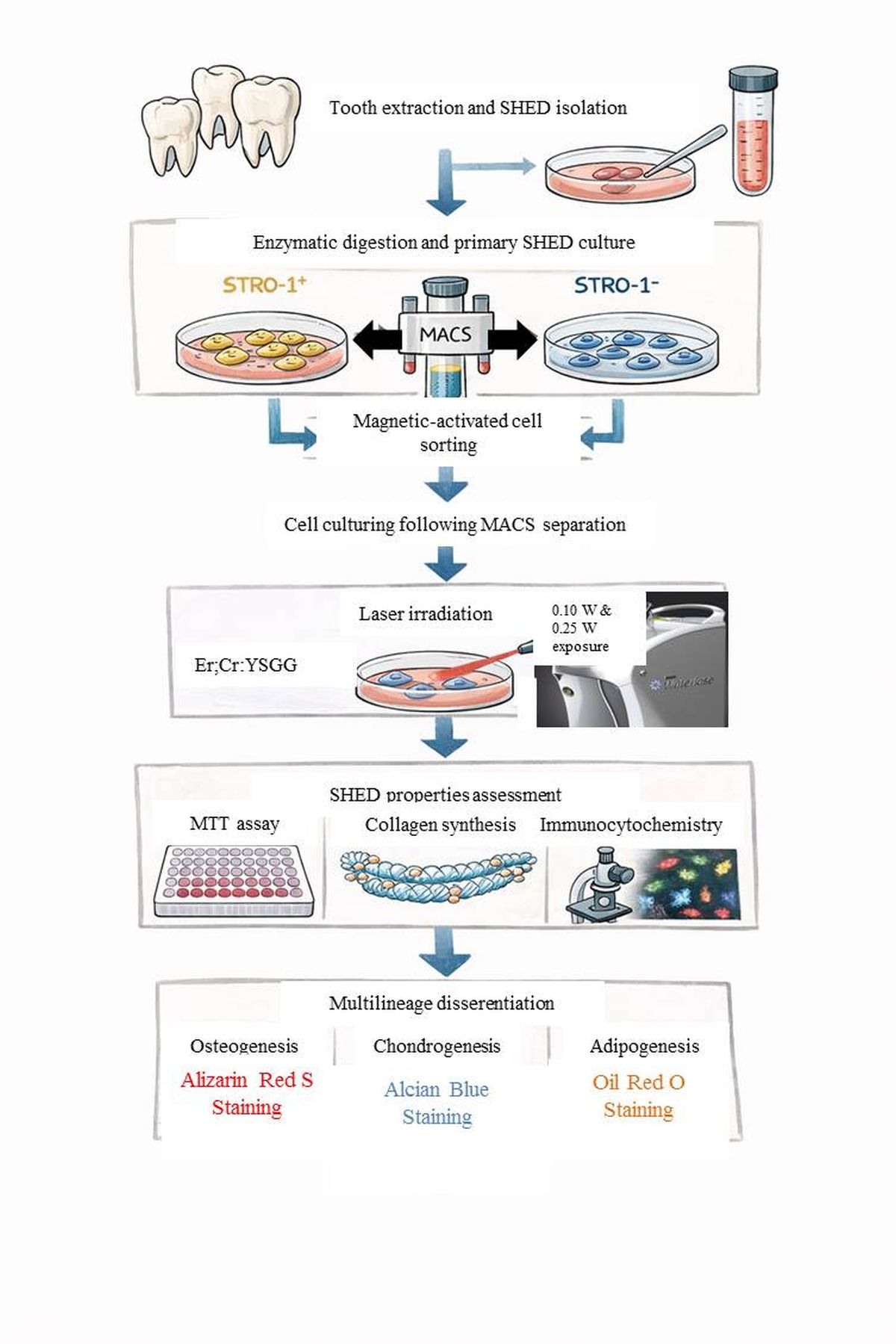

Supplement: Supplementary file 1 [file jfb-17-00138-s001.zip › Figure-S1.jpg]

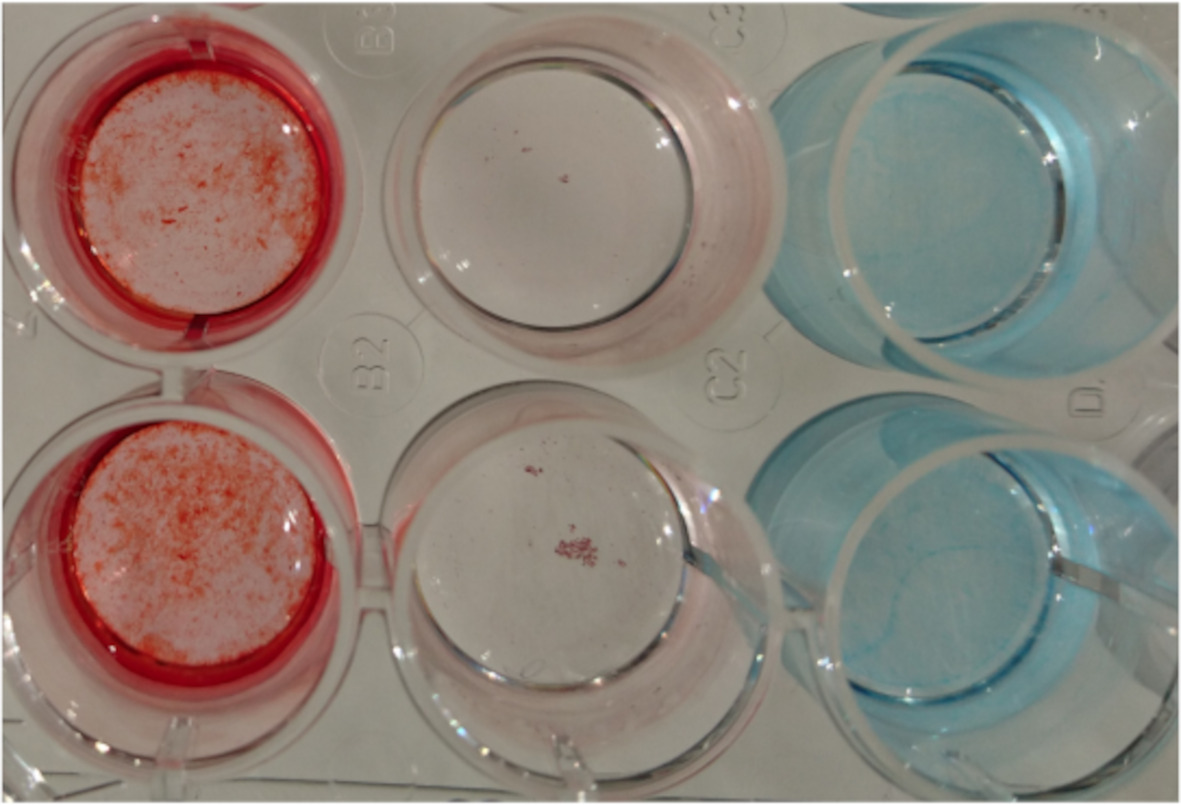

Supplement: Supplementary file 1 [file jfb-17-00138-s001.zip › Supplementary Figure S2 - non-irradiated.jpg]

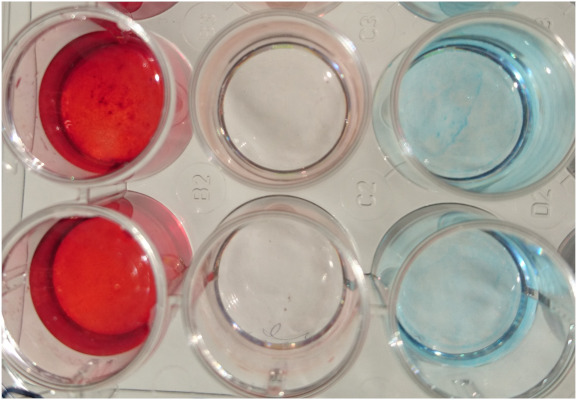

Supplement: Supplementary file 1 [file jfb-17-00138-s001.zip › Supplementary Figure S3 -irradiated.jpg]
